# Optimising nutrients in the culture medium of *Rhodosporidium toruloides* enhances lipids production

**DOI:** 10.1186/s13568-021-01313-6

**Published:** 2021-11-14

**Authors:** Zi Ye, Tongrui Sun, Huoye Hao, Yanling He, Xueyan Liu, Minrui Guo, Guogang Chen

**Affiliations:** School of Food Science and Technology, College of Food, Shihezi University, Shihezi, 832000 China

**Keywords:** Carbon sources, C/N ratio, Lipid production, Nitrogen sources, *Rhodosporidium toruloides*

## Abstract

*Rhodosporidium toruloides* is a useful oleaginous yeast, but lipids production is affected by various factors including nutrients in the culture medium. Herein, the *R-ZL2* high-yield mutant strain was used to investigate the effects of different carbon sources (sucrose, glucose, xylose), nitrogen sources (ammonium sulphate, ammonium nitrate), and C/N ratio on lipids production capacity, get the following conclusion (1) Compared with glucose and xylose, sucrose was a superior carbon source for lipids production; (2) When using ammonium sulphate as the nitrogen source, a C/N ratio of 200:1 achieved the highest biomass, lipids production and lipids content (10.7 g/L, 6.32 g/L and 59%, respectively), and lipids produced under different C/N conditions have potential for biodiesel production (except for C/N = 40 and C/N = 80); (3) When using ammonium nitrate as the nitrogen source, a C/N ratio of 200:1 achieved the highest biomass, lipids production and lipids content (12.1 g/L, 8.25 g/L and 65%, respectively), and lipids produced under different C/N ratio conditions have potential for biodiesel production. Thus, a combination of sucrose and ammonium nitrate was optimal for the lipid accumulation in *R-ZL2*. The findings will lay a foundation for further improving lipids yields.

## Keypoints

- Comparison of lipid production characteristics of *R-ZL2* with different culture condition.
- Increase the lipid production of *R-ZL2* reached 8.25 g/L.
- Ammonium nitrate was nitrogen source, obtained lipid could prepare biodiesel.

## Introduction

Using renewable energy sources to develop energy production and environmentally friendly materials is essential for decreasing fossil fuel use and overcoming growing environmental issues (Yamada et al. 2017). Biodiesel is an important renewable clean energy alternative to fossil fuel-based liquid fuels (Bonturi et al. 2015). However, production of biodiesel has many challenges, especially the increasing price of major feedstocks (plant oils, animal fats and industrial lipids) (Fei et al. 2016; Papadaki et al. 2019). Additionally, arable land is used to grow the required oil crops, resulting in competition between food and fuel crops (Fakas 2017). Therefore, alternative sources for biodiesel production that are economically competitive, renewable, could be produced in large quantities with simple processing, and do not compete with food crop production, are urgently needed.

Microbial lipids obtained from oleaginous microorganisms (e.g., yeast, bacteria and algae) have received considerable attention in recent years because their chemical components are similar to plant-based lipids used for biodiesel production (Christophe et al. 2012; Yen et al. 2015). Using microbial lipids as alternative sources for biodiesel production has many advantages over plant-based lipids, including less pollution, no competition for arable land, and less susceptibility to environmental factors (Sitepu et al. 2014). Various oleaginous microorganisms are capable of accumulating significant amounts of lipids during fermentation under suitable cultivation conditions (Hama et al. 2018). Among these, oleaginous yeasts (e.g., *Candida *sp.,* Rhodosporidium *sp.,* Trichosporon *sp., *Yarrowia *sp. and *Lipomyces *sp.) are considered desirable microbial lipids producers due to their ability to accumulate large quantities of lipids, achieve faster growth, adapt to a wide range of carbon sources, and withstand environmental changes (Liu et al. 2020; Qi et al. 2020).

*Rhodosporidium toruloides* is a promising oleaginous yeast due to its excellent lipids production capacity in terms of high lipids content, yield, and productivity (Saran et al. 2017). *R. toruloides* could grow and accumulate lipids from several carbon sources (Uprety et al. 2017), including glucose, sucrose, xylose (Yamada et al. 2017), glycerol (Uprety et al. 2018), lignocellulosic biomass hydrolysate (Huang et al. 2016), acetic acid and distillery waste-water (Patel et al. 2016; Uprety et al. 2018), and whey from dairy industries (Dulf et al. 2020) to produce microbial lipids. *R. toruloides* has been subjected to mutagenesis to generate high lipids-producing strains, optimisation of culture conditions, and metabolic engineering strategies. However, different strains have differences in adaptability and utilisation of different nutrients. There are relatively few studies on the production of microbial lipids by *R. toruloides* 2.1389 using different nutrients (carbon sources, nitrogen sources, and C/N ratios), and no systematic evaluation of the changes in biodiesel characteristics caused by nutritional differences (Braunwald et al. 2013; Lopes et al. 2020; Saini et al. 2021).

Therefore, the present study aimed to optimise carbon, nitrogen sources and the C/N ratio in the culture medium, to improve the lipids production, biomass, and lipids content by *R-ZL2* (a mutant strain of *R. toruloides* 2.1389 generated by mutagenesis) (Guo et al. 2019). In addition, we determined the fatty acid composition and fatty acid content produced by *R-ZL2* cultured under different nutritional conditions, and evaluated the effects of nutrients on the performance of microbial lipids biodiesel. The findings will lay a foundation for the further application of microbial lipids to the preparation of biodiesel.

## Materials and methods

### Strains, media and cultivation

The *R-ZL2* strain was acquired from our laboratory stocks. In previous research, *R-ZL2* was obtained by mutagenesis and breeding with *R. toruloides* 2.1389 (Guo et al. 2019), and *R. toruloides* 2.1389 was acquired from the China General Microbiological Culture Collection Center (CGMCC). Strains were cultivated in yeast extract peptone dextrose (YEPD) medium (20 g/L peptone, 20 g/L dextrose, 10 g/L yeast extract; 28 °C, 24 h, 150 rpm) and stored at − 80 °C in the presence of glycerol (50% v/v), or in fermentation medium (2 g/L nitrogen source, 1 g/L KH_2_PO_4_, 1 g/L NaCl, 1 g/L MgSO_4_·7H_2_O, 1 g/L CaCl_2_·2H_2_O, 0.5 g/L yeast extract, and 30 g/L carbon sources). The monoclonal cell strain was suspended in 5 mL YEPD medium in φ16 × 125 mm^2^ test tubes, transferred to 250 mL flasks containing 100 mL of fermentation medium, and incubated in a rotatory shaker (28 °C, 150 rpm).

### Fermentation conditions

In the first phase of the experiment, ammonium sulphate was used as nitrogen source, different concentrations of glucose, sucrose and xylose were used as fermentation carbon sources (sugar concentrations were 1%, 2%, 3%, 4% and 5%) to study the effects on biomass, lipids production, sugar consumption and lipids yield of the *R-ZL2* strain, and thereby determined the best carbon source and the optimal quantity.

In the second stage, the optimal carbon source and quantity determined in the first stage were employed during fermentation, and ammonium sulphate was used as nitrogen source to study the influence of C/N ratio on biomass, lipids production, sugar consumption, lipids yield and fatty acid production.

In the third stage, ammonium nitrate was used as nitrogen source to study the influence of C/N ratio on biomass, lipids production, sugar consumption, lipids yield and fatty acid production.

### Determination of biomass

In order to estimate the yeast dry biomass (dry cell mass), 10 mL of culture broth was centrifuged (8000*g*/min, 10 min). The supernatant was discarded and wet cells were washed three times with sterile water. The total dry cell weight was determined after drying at 70 °C for 12 h. All experiments were done in triplicate, the results were averaged.

### Determination of lipids production

Lipid production was determined by the vanillin phosphate method (Yamada et al. 2017). First, 2 mL of culture broth was centrifuged (8000*g*/min, 5 min). The supernatant was discarded, wet cells were washed twice with sterile water, and the volume was made up to 1 mL with sterile water. A 100 µL volume of yeast suspension (100 µL of sterile water as a blank control) was placed in a glass test tube with 2 mL of concentrated sulphuric acid, boiled in a water bath for 10 min, cooled for 5 min at room temperature, and 5 mL of vanillin phosphate solution was added and incubated at 37 °C for 20 min. After cooling at room temperature and transferring to a centrifuge tube, samples were centrifuged (4000*g*/min, 5 min), and the supernatant was analysed by spectrophotometry (Shimadzu Instruments Co., Ltd., China) at 530 nm. Lipid production was calculated based on a standard curve (Y = 0.2891X + 0.01119, R^2 ^= 0.9995), expressed the lipid yield in g-lipid/L-fermentation liquid. If there was a dilution relationship during the operation, multiply it by the corresponding dilution factor in the final calculation. All experiments were done in triplicate, the results were averaged.

### Determination of residual sugar content

When determining the concentration of sucrose, we first hydrolyze sucrose to reducing sugar with hydrochloric acid, and then determine the content of residual sugar by 3,5-dinitrosalicylic acid (DNS) method (Zhang et al. 2019). First, 5 mL culture broth was centrifuged (8000*g*/min, 5 min), and 20 µL of supernatant was placed in a 25 mL colorimetric tube with 2 mL of distilled water and 3 mL of DNS, boiled for 10 min, quickly cooled on ice, diluted to 25 mL, mixed by shaking, and the absorbance at 520 nm was measured by spectrophotometer. All experiments were done in triplicate, the results were averaged.

### Determination of lipids content

The lipids concentration was determined by the methanol-chloroform extraction method with some modifications (Namitha et al. 2021). First, 10 mL of culture broth was centrifuged (8000*g*/min, 5 min). The supernatant was discarded and wet cells were washed three times with sterile water. Wet cells were then transferred to a 20 mL glass tube and 2 mL of 4 M HCl was added and incubated at 78 °C in a water bath for 1 h, then cooled to room temperature. Next, 4 mL of 1:1 (v/v) methanol: chloroform was added and vortexed for 2 min, samples were centrifuged (8000*g*/min, 5 min), and the chloroform layer was withdrawn using a pipette and collected in a new centrifuge tube. The extraction was repeated by adding 2 mL chloroform into the remaining layer, rigorously shaking for 2 min, centrifuging, and collecting the chloroform layer. The chloroform layers from both the extraction stages were combined and washed with 4 mL of 0.1% NaCl. Finally, the reaction mixture was centrifuged (8000*g*/min, 5 min), the chloroform layer was collected into a pre-weighed vial, dried at 70 °C for 12 h, and the total lipids content was quantified by the gravimetric method. All experiments were done in triplicate, the results are averaged.

### Determination of fatty acid composition

Fatty acid composition was analysed by gas chromatography-mass spectrometry (GC/MS) using an Agilent 5975 series MSD and an Agilent 7890 A instrument equipped with an HP-5 column (30 m × 0.25 mm, film thickness 0.25 m; HP). The program used for GC/MS analysis was as follows: 140 °C for 2 min, heating to 180 °C at a rate of 5 °C/min holding at 180 °C for 5 min, heating to 230 °C at 5 °C/min, holding at 230 °C for 6 min with electron ionisation of 70 eV (Guo et al. 2019). Fatty acid composition of microbial lipids produced under different nutritional conditions were statistically analysed by t-tests and *p ≤* 0.05 was considered to indicate statistical significance.

### Evaluation of biodiesel properties

The physical and chemical properties (viscosity, specific gravity, turbidity, cetane number, iodine value and high calorific value) of biodiesel fuel were roughly evaluated by lipids composition according to a method described previously (Tanimura et al. 2014).

### Statistical analysis

All data were expressed as the mean ± standard deviation (SD). Origin 2021 software was used to make charts. SPSS statistics software was used to analyze the statistical significance; *p ≤* 0.05 was considered to indicate statistical significance.

## Results

### Different carbon sources on the lipids production characteristics of *R-ZL2*

Strains ccould adapt to different carbon sources and exhibit distinct substrate preferences (Lin and Wu 2015). To investigate the ability of *R-ZL2* to utilise and assimilated different carbon sources, we compared the biomass, lipids production, sugar consumption, and lipids yield of strains cultured at 96 h in different concentrations of sucrose, glucose and xylose to determine the optimal carbon source and the best dosage.

**Fig. 1:**
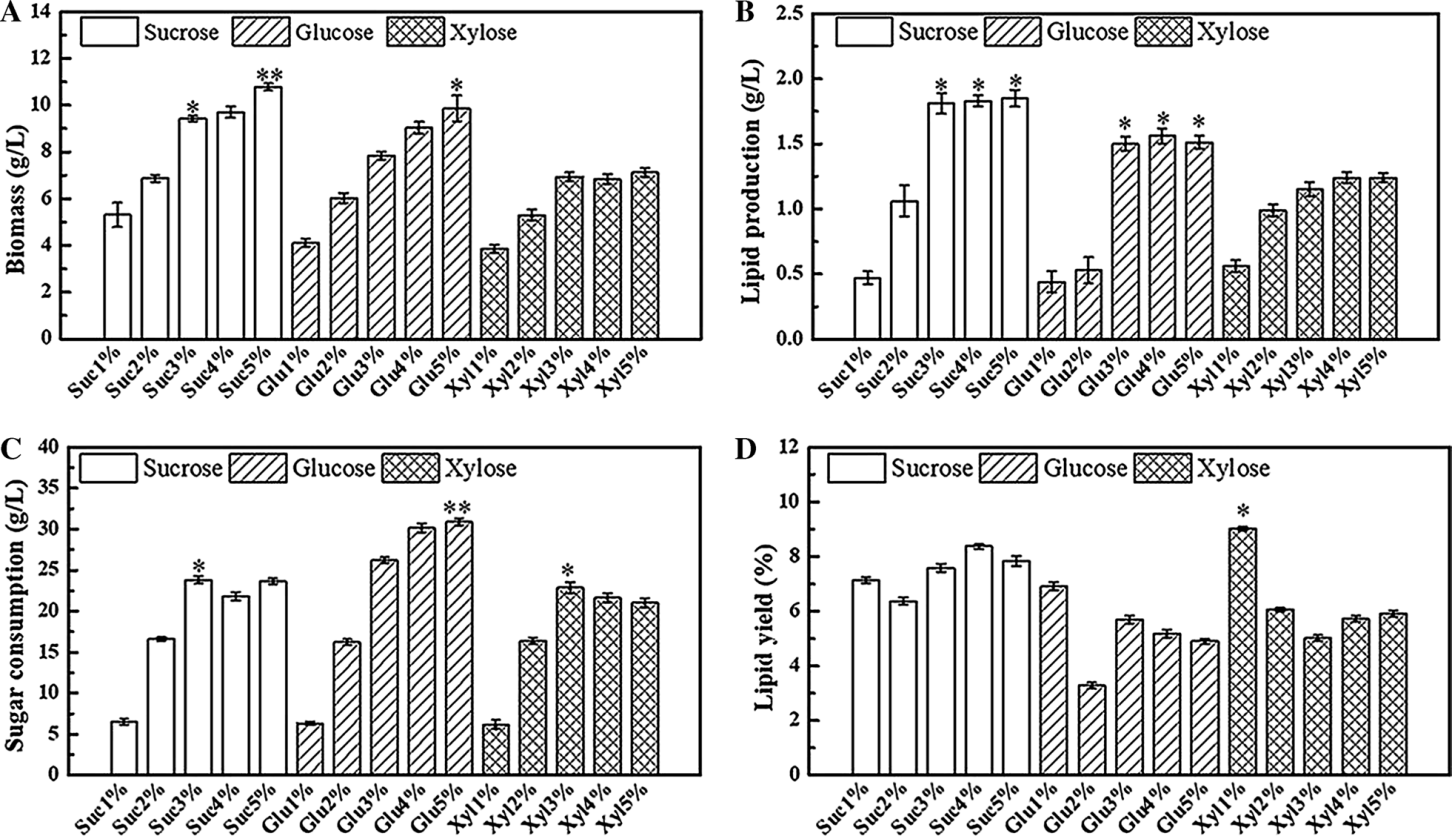
Effects of different mass fractions of carbon sources on yeast. **A** Impact on biomass, **B** impact on lipid production, **C** impact on sugar consumption, **D** impact on lipid yield. The experimental data is measured at the 96th h of culture. Data are presented as the average of three independent experiments. Error bars represent means ± standard deviation. Significant differences were calculated using the t test (**p < 0.01, *p < 0.05)

Our results shown that the biomass of strains increased with increasing sugar concentration for sucrose and glucose (Fig. 1a). When the concentration of sucrose and glucose was 5%, the biomass reached the maximum value (10.77 g/L and 9.85 g/L, respectively), suggested that the carbon sources satisfied the growth needs of the strains. Comparing the effects of different carbon sources with the same mass fraction on the biomass accumulation of strains, sucrose was found to be most effective. When the xylose concentration was 1−3%, the biomass increased with increasing sugar mass fraction, but when the xylose concentration was > 3%, the biomass did not increase significantly (*p* > 0.05). Therefore, sucrose as the carbon source was most conducive to biomass and lipid accumulation in *R-ZL2*, because sucrose is a disaccharide, which provides more energy than glucose, and when the culture medium was sterilized, sucrose is more stable and glucose is easily carbonized, xylose is a non-fermentable carbon source, strain’s utilization rate for it was low and cannot be fully utilized within the same fermentation time.

There was a correlation between lipids production and the concentration of the carbon source (Fig. 1b). When the amount of sucrose added was 4% and 5%, there was no significant difference in lipids production (*p* > 0.05), indicating that excess carbon source was not effectively converted into lipids by the strains. The fermentation trends for glucose and xylose were similar, when the carbon source was < 3%, accumulation of lipids and biomass was low, and when the sugar concentration was 3−5%, there was no significant difference in lipids production (*p* > 0.05), indicating that excess carbon source was not used for lipids accumulation. It could be concluded that carbon source concentrations > 3% were optimal for accumulation of lipids, and sucrose was the best sugar substrate.

Sugar consumption reflects a strain’s ability to transform and utilise carbon sources. As shown in Fig. 1c, when sucrose addition was < 3%, sugar consumption was low, possibly due to carbon source inhibition; when sucrose addition was > 3%, the difference in sugar consumption was not significant (*p* > 0.05), indicating that sugar consumption maybe limited within the specified time. Compared with sucrose, strains were better able to consume glucose, and sugar consumption of different concentrations of glucose was higher than sucrose fermentation. The ability to consume xylose was similar to sucrose.

The lipids yield is the amount of lipids produced per unit mass of carbon source, which could further verify whether the carbon source is effectively converted. As shown in Fig. 1d, when sucrose was the carbon source, the lipids yield with 4% was the highest; when glucose was the carbon source, the lipids yield with 3% was the highest; when xylose was the carbon source, the lipids yield with 5% was the highest.

In summary, the preference of *R-ZL2* for three carbon sources was ordered sucrose > glucose > xylose. In addition, sufficient carbon is needed for growth, but excess carbon sources do not promote lipids production. Therefore, we chose 4% sucrose as the carbon source for follow-up experiments.

### Different C/N ratios on the lipids production characteristics of *R-ZL2* with ammonium sulphate as the nitrogen source

Ammonium sulphate was widely used in the cultivation of oleaginous yeast, providing the nitrogen source for yeast growth and lipids production. However, the concentration of the nitrogen source had a great influence on both the growth and lipids production of the strains. Therefore, we used 4% sucrose as the carbon source and ammonium sulphate as the nitrogen source to study the effect of different C/N ratios (20:1, 40:1, 80:1, 160:1, 200:1 and ∞ (∞ indicate that no nitrogen source was added to the medium)) on the lipids production characteristics of *R-ZL2*.

**Fig. 2:**
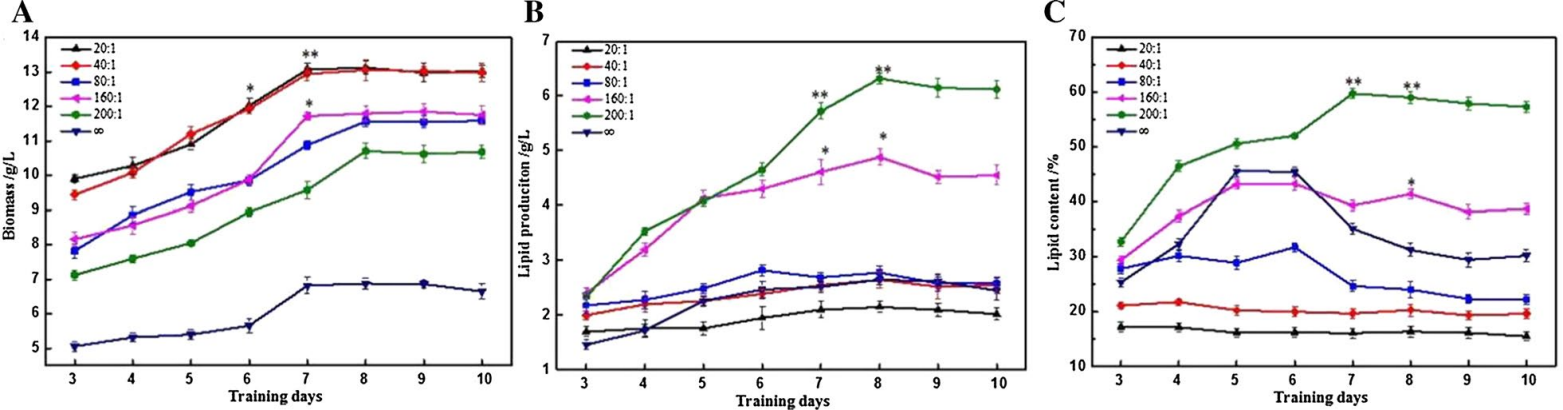
Changes of biomass (**A**), lipid production (**B**), lipid content (**C**) of *R-ZL2* in different C/N ratios (the 4% sucrose as the carbon source, ammonium sulfate as the nitrogen sources). Data are presented as the average of three independent experiments. Error bars represent means ± standard deviation. Significant differences were calculated using the t test (**p < 0.01, *p < 0.05)

With increasing fermentation duration, the biomass of strains in media with different C/N ratios displayed an upward trend, then tended to be stable, while further extension of the fermentation time did not change the biomass significantly (*p* > 0.05; Fig. 2a). When the C/N ratio was ∞, the minimum biomass was 6.8 g/L, when the C/N ratio was 20:1 and 40:1, the maximum biomass was 13.07 g/L and 12.94 g/L, respectively. The results shown that the nitrogen source content in the range of 1−2 g/L was more conducive to biomass accumulation.

The lipids production of strains in different C/N ratio media gradually increased with increasing fermentation duration until the 8th day of fermentation, at which point lipids production reached a peak (Fig. 2b). When the C/N ratio was 20:1, lipids production was the lowest (2.15 g/L); when the C/N ratio was 160:1 and 200:1, lipids production was the highest (4.88 g/L and 6.32 g/L, respectively). From this, we concluded that 4% sucrose as the carbon source, ammonium sulphate as the nitrogen source, and a C/N ratio of 200:1 were the optimal conditions for lipids production.

The effect of different C/N ratios on the lipids content of the strains shown in Fig. 2c. During the entire fermentation process, the lipids content of the experimental group with a C/N ratio of 200:1 was consistently significantly higher than other groups (*p* < 0.05), and the lipids content reached the maximum value on the 7th day (59.73%), indicating that the strains had a strong ability to produce intracellular lipids. With a C/N ratio of 160:1, the lipids content was 43.26%; and when the C/N ratios was 20:1, the lipids content was only 16%, significantly lower than other groups (*p* < 0.05). When the C/N ratio was ∞, the lipids content of the strains was increased to a certain extent, but lipids content is not the highest. The above results indicated that the nitrogen source was indispensable for lipids accumulation of the strains, and effective control of the C/N ratio important for lipids accumulation.

### Different C/N ratios on the lipids production characteristics of *R-ZL2* with ammonium nitrate as the nitrogen source

Ammonium nitrate is a good nitrogen source. In order to investigate the effect of ammonium nitrate on yeast growth and lipids accumulation, and to compare with yeast cultured with ammonium sulphate as the nitrogen source, we explored using 4% sucrose as the carbon source, ammonium nitrate as the nitrogen source, and assessed the effect of different C/N ratios on the lipids production characteristics of yeast.

**Fig. 3:**
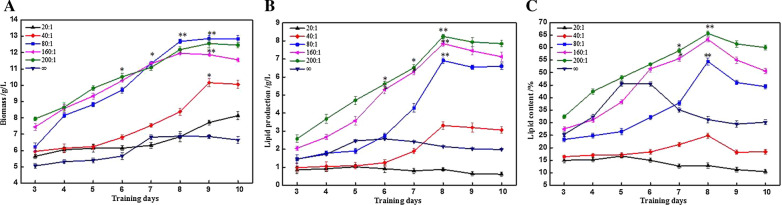
Changes of biomass (**A**), lipid production (**B**), lipid content (**C**) of *R-ZL2* in different C/N ratios (the 4% sucrose as the carbon source, ammonium nitrate as the nitrogen sources). Data are presented as the average of three independent experiments. Error bars represent means ± standard deviation. Significant differences were calculated using the t test (**p < 0.01, *p < 0.05)

With increasing fermentation duration, the biomass of yeast increased continuously (Fig. 3a). The biomass was the lowest when the C/N ratio was ∞, and the biomass reached the maximum value on the 7th day (6.82 g/L). When the C/N ratio was 20:1, the biomass was slightly higher than that when the C/N ratio was ∞, but lower than other experimental groups. When the C/N ratio was 40:1, the biomass reached the maximum value on the 9th day (10.16 g/L). The biomass of the experimental groups with a C/N ratio of 80:1, 160:1 and 200:1 was always higher than previous three groups, at the end of fermentation, the biomass of them was 12.35 g/L, 12.56 g/L and 11.88 g/L, respectively. Therefore, when ammonium nitrate as the nitrogen source, effective control of the amount of nitrogen source added could promote the accumulation of biomass. Comparing the biomass with a C/N ratio of 200:1 and 20:1 shown that the biomass was increased by 1.8-fold. When ammonium sulphate as the nitrogen source, the biomass was the highest when the C/N was 20:1 (13.07 g/L). The above results indicated that the strains have differences in their utilisation of different nitrogen sources, and there were significant differences in the biomass of strains cultured with different C/N ratios.

C/N ratio of 200:1 achieved the highest lipids production, significantly higher than other groups during the entire fermentation stage (*p* < 0.05, Fig. 3b), and reached the maximum value on the 8th day (8.25 g/L). By comparison, a C/N ratio of 160:1 reached 7.85 g/L, a C/N ratio of 80:1 and 40:1 achieved intermediate amounts of lipids production, and a C/N of 20:1 recorded the lowest lipids production. When the C/N was ∞, lipids production was slightly higher than 20:1, but significantly lower than other experimental groups (*p* < 0.05). The above results shown that when ammonium nitrate as the nitrogen source, increasing the C/N ratio could promote the accumulation of lipids, but when the nitrogen source was not added at all, lipids production was not favoured. Thus, the optimal C/N ratio was determined to be 200:1. This result was consistent with the results for ammonium sulphate as the nitrogen source. When the C/N ratio was 200:1, lipids production using ammonium sulphate or ammonium nitrate as the nitrogen source was 6.32 g/L and 8.25 g/L, respectively. Therefore, ammonium nitrate was more advantageous when mixed with sucrose to culture *R-ZL2* to produce lipids.

The effects of different C/N ratios on the lipids content of strains shown in Fig. 3c. When the C/N ratio was 200:1, the lipids content reached the maximum value on the 8th day (65.64%). When the C/N ratio was 160:1, the lipids content was slightly lower than ratio of 200:1, and the maximum value was also reached on the 8th day. When the C/N ratio was 20:1, the lipids content of the strains was the lowest (~ 15%). When the C/N ratio was ∞, the lipids content of the strains was slightly higher than 20:1, but significantly lower than when the C/N was 200:1, 160:1 or 80:1 (p < 0.05). These results shown that using an appropriate carbon source and C/N ratio could increase the lipids content.

### Evaluation of the performance of biodiesel in the fatty acid composition of lipids produced by *R-ZL2* under different nutrient conditions

Using sucrose as the carbon source and ammonium sulphate as the nitrogen source, we determined the fatty acid composition of lipids produced by *R-ZL2* cultured with different C/N ratios (Table 1). Fatty acids produced by yeast cultured with different C/N ratios were C14:0, C16:0, C18:0, C18:1, C18:2 and C18:3, among which C16:0, C18:1 and C18:2 was the most abundant. With increasing C/N ratio in the medium, the ability of yeast to produce C16:0 gradually increased. When the C/N ratio was 160:1, the accumulated C16:0 content was the highest, and continued to increase the C/N ratio, the change in C16:0 was not significant (*p* > 0.05). In addition, with an increase in C/N ratio in the medium, the ability of yeast to produce C18:1 weakened. When the C/N ratio was 20:1, the content of C18:1 was the highest. Continued to increase the C/N ratio, the content of C18:1 was significantly decreased (*p* < 0.05). C18:2 as most abundant when the C/N ratio was 40:1. The C18:3 content was the lowest when the C/N ratio was 20:1. The abundance of C18:3 did not differ significantly between different culture conditions. In summary, a change in the C/N ratio in the medium could alter the fatty acid composition of the lipids produced.

**Table 1:** Changes of fatty acid content of *R-ZL2* in different C/N ratios

C: 4 % sucrose N: ammonium sulfate	C14:0 (% w/w)	C16:0 (% w/w)	C18:0 (% w/w)	C18:1 (% w/w)	C18:2 (% w/w)	C18:3 (% w/w)	Total fatty acid (g/L)
C/N = 20:1	0.52 ± 0.05	13.92 ± 0.11	9.02 ± 0.15	42.33 ± 1.23	28.49 ± 1.11	5.72 ± 0.53	7.769
C/N = 40:1	0.90 ± 0.06	17.55 ± 0.13	5.57 ± 0.41	30.04 ± 1.26	32.31 ± 1.32	13.64 ± 1.60	6.0769
C/N = 80:1	1.18 ± 0.09	21.64 ± 1.3	6.49 ± 0.44	29.73 ± 1.22	27.37 ± 1.26	13.59 ± 1.65	4.638
C/N = 160:1	1.44 ± 0.11	24.72 ± 1.5	8.00 ± 0.83	33.18 ± 1.25	19.26 ± 1.06	13.40 ± 1.06	2.436
C/N = 200:1	1.32 ± 0.10	24.37 ± 1.4	7.81 ± 0.69	31.43 ± 1.82	20.17 ± 1.14	14.91 ± 1.02	1.761
C/N = ∞	1.21 ± 0.09	24.00 ± 1.3	9.73 ± 0.93	34.68 ± 1.95	18.07 ± 0.97	12.31 ± 0.98	0.924

The fatty acid results when using sucrose as the carbon source and ammonium nitrate as the nitrogen source shown in Table 2. The identified fatty acids were composed of C14:0, C16:0, C18:0, C18:1, C18:2 and C18:3, and the most abundant were C16:0, C18:1 and C18:2 (as observed for ammonium sulphate as the nitrogen source). C16:0 was the most abundant when the C/N ratio was 80:1. C18:1 had the highest content when the C/N ratio was 40:1, and the lowest content when the C/N ratio was 80:1. Levels of C18:2 was the highest when the C/N ratio was 20:1, and levels decreased with increasing C/N ratio. The content of C18:3 was the lowest when the C/N ratio was 20:1, but it did not differ much between other conditions, indicating minimal effect on the synthesis of C18:3.

**Table 2:** Changes of fatty acid content of *R-ZL2* in different C/N ratios

C: 4 % sucrose N: ammonium nitrate	C14:0 (% w/w)	C16:0 (% w/w)	C18:0 (% w/w)	C18:1 (% w/w)	C18:2 (% w/w)	C18:3 (% w/w)	Total fatty acid (g/L)
C/N = 20:1	0.97 ± 0.06	17.55 ± 0.62	7.32 ± 0.23	44.12 ± 2.03	26.03 ± 0.95	4.01 ± 0.16	4.88
C/N = 40:1	1.12 ± 0.15	21.74 ± 0.75	6.87 ± 0.31	45.13 ± 1.79	20.95 ± 0.61	4.18 ± 0.46	4.96
C/N = 80:1	1.62 ± 0.18	26.98 ± 0.56	9.19 ± 0.46	41.71 ± 2.43	14.88 ± 0.39	5.62 ± 0.38	6.35
C/N = 160:1	1.44 ± 0.09	25.91 ± 0.34	10.06 ± 0.64	43.78 ± 1.95	12.58 ± 0.98	6.24 ± 0.61	5.82
C/N = 200:1	1.42 ± 0.16	25.61 ± 0.49	10.08 ± 0.43	43.48 ± 1.63	12.47 ± 0.69	6.94 ± 0.42	5.32
C/N = ∞	1.07 ± 0.09	24.19 ± 0.65	11.57 ± 0.35	43.71 ± 1.25	12.19 ± 0.93	7.27 ± 0.36	1.32

We evaluated whether the lipids produced under different C/N ratios could be used as biodiesel based on the content of each fatty acid (Table 3), through comparison of viscosity, specific gravity, turbidity, cetane number, iodine value and calorific value. When the carbon source was 4% sucrose and the nitrogen source was ammonium sulphate, the unsaturation of the lipids produced by yeast at C/N ratios of 40:1 and 80:1 was the highest, resulting in iodine values greater than the maximum limit of the national standard, which is unsuitable for the production of biodiesel.

**Table 3:** Comparison of the properties of biodiesel for lipid by *R-ZL2* fermented with different C/N ratios

C: 4% sucrose N: ammonium sulfate	Viscosity (mm/s^2^)	Specific gravity	Cloud point (°C)	Cetane number	Iodine number	Higher heating value (MJ/kg)
C/N = 20:1	4.47	0.8790	4.43	55.10	99.33	40.58
C/N = 40:1	4.35	0.8801	1.89	53.83	113.54	40.92
C/N = 80:1	4.42	0.8795	3.27	54.52	105.85	40.74
C/N = 160:1	4.50	0.8788	5.05	55.41	95.93	40.50
C/N = 200:1	4.47	0.8790	4.43	55.11	99.35	40.58
C/N = ∞	4.53	0.8788	5.60	55.69	92.85	40.43
S50	1.9–6.0	0.82–0.9	−	49 (minimum)	101 (maximum)	−
ASTM D6751	1.9–6.0	−	−	47 (minimum)	93 (maximum)	−
EN 14,214	3.5–5.0	0.86–0.9	−	51 (minimum)	120 (maximum)	−

When 4% sucrose as the carbon source, ammonium sulfate as the nitrogen sources comparison of the properties of biodiesel for lipid by *R-ZL2* fermented with different C/N ratios with those of Chinese, U.S. and EU biodiesel standards

Using 4% sucrose as the carbon source (Table 4), ammonium nitrate as the nitrogen source, and different C/N ratios, the lipids produced by yeast were found to be suitable for the production of biodiesel. In addition, we found that the fatty acids produced by yeast under different C/N ratios contained 60−70% unsaturated fatty acids and 25−40% saturated fatty acids. This shown that *R-ZL2* has a strong ability to produce unsaturated fatty acids when using ammonium nitrate as the nitrogen source.

**Table 4:** Comparison of the properties of biodiesel for lipid by *R-ZL2* fermented with different C/N ratios

C: 4% sucrose N: ammonium nitrate	Viscosity (mm/s^2^)	Specific gravity	Cloud point (°C)	Cetane number	Iodine number	Higher heating value (MJ/kg)
C/N = 20:1	4.52	0.8786	5.54	55.66	93.19	40.44
C/N = 40:1	4.58	0.8781	6.69	56.24	86.77	40.29
C/N = 80:1	4.65	0.8775	8.20	56.99	78.40	40.09
C/N = 160:1	4.65	0.8774	8.29	57.03	77.89	40.08
C/N = 200:1	4.64	0.8775	8.08	56.93	79.08	40.10
C/N = ∞	4.64	0.8775	7.99	56.88	79.56	40.12
S50	1.9–6.0	0.82–0.9	−	49 (minimum)	101 (maximum)	−
ASTM D6751	1.9–6.0	−	−	47 (minimum)	93 (maximum)	−
EN 14,214	3.5–5.0	0.86–0.9	−	51 (minimum)	120 (maximum)	−

When 4% sucrose as the carbon source, ammonium nitrate as the nitrogen sources comparison of the properties of biodiesel for lipid by *R-ZL2* fermented with different C/N ratios with those of Chinese, U.S. and EU biodiesel standards)

## Discussion

The growth of lipids-producing microorganisms and some metabolic activities are affected by the different carbon sources and the amount of carbon source added in the medium (Gong et al. 2019). Different carbon sources such as glucose, sucrose, xylose and glycerol have been used in the cultivation of different lipids-producing microorganisms. However, due to the growth specificity of microorganisms, the preference for substrates, and the limitations of culture conditions, different microorganisms are affected differently by different carbon sources. In the present study, we investigated the utilisation of different carbon sources (sucrose, xylose and glucose) by *R-ZL2*. Glucose has been used as the main nutrient in the culture medium to grow numerous lipids-producing microorganisms. Studies have shown that *Chlorella sorokiniana* and *Scenedesmus obliquus* could use glucose as the carbon source for rapid growth (Shen et al. 2018; Wang et al. 2020), *Rhodotorula glutinis* could use glucose to simultaneously produce microbial lipids and carotenoids (Gong et al. 2019). The above studies proved the positive effect of glucose on microbial growth and metabolism. Sucrose, another carbon source widely used in the cultivation of lipids-producing microorganisms, could also promote the production of microbial lipids. Lin and Wu (2015) used sucrose as the carbon source for mixed culture of *C. sorokiniana* and found that when 5% sucrose achieved the highest growth rate and lipids content for *C. sorokiniana*. Similarly, in the present study, when sucrose was the carbon source, 5% sucrose achieved the maximum *R-ZL2* biomass (10.77 g/L). Xylose is a low-cost carbon source that could also promote growth and lipids production in *S. obliquus*, Song and Pei (2018) proved that when xylose was added at 4 g/L, achieved the highest specific growth ratethe accumulation of biomass and lipids. In the present study, when the xylose concentration was > 3%, the biomass of *R-ZL2* did not continue to increase, but instead plateaued. This indicateed that when xylose was the carbon source, only a certain concentration range could promote the growth of *R-ZL2.*

The nitrogen source is one of the key factors affecting the growth of lipids-producing microorganisms and lipids accumulation (Chiu et al. 2015). The challenge faced by many researchers is how to select the optimal nitrogen source and suitable concentration for the growth and lipids accumulation of oleaginous microorganisms. Studies have found that optimising nitrogen sources could effectively increase lipids production during the cultivation of different lipids-producing microorganisms (Gonzalez-Garcinuno et al. 2014). By optimising the nitrogen and carbon sources in the medium, Isleten-Hosoglu et al. (2012) determined that *C. saccharophila* could achieved maximum biomass when the medium contained 1 g/L bacterial peptone and 20 g/L glucose, 7.7-fold higher than the control group. Wu et al. (2013) used ammonium nitrate, potassium nitrate and ammonium chloride as nitrogen sources to cultivate microalgae, and found that lipids productivity and biomass concentration were the highest (29.2 mg/L/day and 650 mg/L respectively) when potassium nitrate was the nitrogen source. In addition, Saini et al. (2021) applied sulphate as nitrogen source to cultivate oleaginous yeast, and the results shown that an appropriate sulphate concentration could effectively stimulate oleaginous yeast to accumulate large quantities of intracellular lipids. Ammonium sulphate and ammonium nitrate are the broad-spectrum nitrogen sources for the cultivation of lipids-producing microorganisms. Usually, the different proportion of nitrogen will have a significant impact on the growth and lipids-producing ability of the strains. When analysing the effects of nitrate and ammonium on microalgae metabolism, Campos et al. (2014) found that ammonium nitrate was more beneficial as the nitrogen source to increase lipids production and biomass of strains, because ammonium nitrate contains both nitrate and ammonium, thus avoiding possible saturation in the transporter (Gonzalez-Garcinuno et al. 2014). Our current results also confirmed that ammonium nitrate was more conducive to the accumulation of *R-ZL2* biomass and lipids than ammonium sulphate.

Carbon and nitrogen sources are important nutrients for the growth of strains. Numerous studies have shown that different carbon and nitrogen sources have different effects, the accumulation of lipids in microorganisms is stimulated by high carbon sources and low nitrogen sources in the medium, and there was a positive correlation between lipids production and C/N ratio (Saran et al. 2017). An appropriate C/N ratio is the key to maximising the growth and lipids production capacity of strains. Khichi et al. (2019) shown that *Botryococcus braunii* achieved the maximum biomass accumulation and lipids production when the C/N ratio was 29:1 (1.11 g/L/day and 0.390 g/L/day, respectively). Matsakas et al. (2015) shown that an appropriate C/N ratio could stimulate lipids-producing microorganisms to accumulate more microbial lipids. Of course, different lipids-producing microorganisms have preferences for different C/N ratios, and this must be adjusted according to their growth characteristics in order to achieve optimal production of microbial lipids.

Furthermore, the C/N ratio in the medium could also affect the fatty acid composition of microbial lipids, which determines whether they could be used in the production of biodiesel. Gao et al. (2019) study found that a higher initial C/N ratio increases the ratio of saturated fatty acids (C16:0 and C18:0) in microalgae lipids production; when Ramanna et al. (2014) cultured *C. sorokiniana* with urea as the nitrogen source, the results shown that the carbon chain was composed of fatty acids ranging from C12:0 to C24:0. A large amount of polyunsaturated fatty acids (PUFA) affects the oxidative stability of biodiesel, and as the raw material of biodiesel, the composition of saturated fatty acids of microbial lipids is critical. Adjusting the C/N ratio in the medium to strictly control the ratio of saturated fatty acids and unsaturated fatty acids could result in products amenable for biodiesel usage.

Different C/N ratios could have significant effects on the growth, lipids accumulation and fatty acid composition of *R-ZL2*. Therefore, optimising the culture conditions of *R-ZL2* was practical significance for improving lipids production and promoting biodiesel production.

In conclusions, our results shown that different carbon sources (sucrose, glucose and xylose) could be applied in lipids-producing of *R. toruloides* mutant strain *R-ZL2*. Yeast cultured with sucrose as the carbon source displayed the strongest lipids-producing ability, followed by glucose, and finally xylose.

When sucrose and ammonium sulphate as the carbon and nitrogen sources, respectively, the optimal C/N ratio was 200:1, for which the highest biomass, lipids yield and lipids content values were 10.7 g/L, 6.32 g/L and 59%, respectively. When the C/N was 40:1 and 80:1, the unsaturation of microbial lipids was high, which led to a higher iodine value than the maximum limit of the national standard, making it unsuitable for the preparation of biodiesel. Other C/N ratios could be used for fermentation production of lipids in the preparation of biodiesel.

With ammonium nitrate as the nitrogen source, the optimal C/N ratio was also 200:1, and the highest biomass, lipids yield and lipids content values were 12.1 g/L, 8.25 g/L and 65%, respectively. The fatty acids of microbial lipids produced by different C/N ratios contained 60−70% unsaturated fatty acids and 25−40 % saturated fatty acids. According to the evaluation of biodiesel characteristics, different C/N ratios could achieve lipids that are suitable for the preparation of biodiesel.

After comprehensive investigation of parameters related to lipids synthesis, the optimal conditions for lipids production by *R-ZL2* were found to be 4% sucrose as the carbon source, ammonium nitrate as the nitrogen source, and a C/N ratio of 200:1.

## Data Availability

The data that support the findings of this study are available on request from the corresponding author. The data are not publicly available due to privacy or ethical restrictions.

## Abbreviations

YEPD: Yeast extract peptone dextrose
FM: Fermentation medium
CGMCC: China General Microbiological Culture Collection Center
DNS: 3,5-Dinitrosalicylic acid
